# Ameliorative Effects of Dietary Ellagic Acid Against Severe Malaria Pathogenesis by Reducing Cytokine Storms and Oxidative Stress

**DOI:** 10.3389/fphar.2021.777400

**Published:** 2021-12-09

**Authors:** Shilpa Mohanty, Amit Chand Gupta, Anil Kumar Maurya, Karuna Shanker, Anirban Pal, Dnyaneshwar Umrao Bawankule

**Affiliations:** ^1^ In-vivo Testing Laboratory, Molecular Bioprospection Department, CSIR-Central Institute of Medicinal and Aromatic Plants, Lucknow, India; ^2^ Academy of Scientific and Innovative Research (AcSIR), Ghaziabad, India; ^3^ Analytical Chemistry Department, CSIR-Central Institute of Medicinal and Aromatic Plants, Lucknow, India

**Keywords:** ellagic acid, malaria, inflammation, cytokine, storms, oxidative stress, mice

## Abstract

Ellagic acid (EA), a fruit- and vegetable-derived flavonoid, has been reported for multiple pharmacological activities, which encouraged us to examine its useful effect in severe malaria pathogenesis, especially malaria-induced cytokine storms and oxidative stress linked to damage in major organs. Malaria was induced by injecting *Plasmodium berghei*–infected RBCs intraperitoneally into the mice. EA was given orally (5, 10, and 20 mg/kg) following Peter’s 4-day suppression test. EA exhibited the suppression of parasitemia, production of inflammatory cytokine storms and oxidative stress marker level quantified from vital organs significantly and an increase in hemoglobin, blood glucose, and mean survival time compared to the vehicle-treated infected group. EA administration also restored the blood–brain barrier integrity evidenced through Evans blue staining. Furthermore, we demonstrated the protecting effect of EA in LPS-induced inflammatory cytokine storms and oxidative stress in glial cells. The present study conclude that ellagic acid is able to alleviate severe malaria pathogenesis by reducing cytokine storms and oxidative stress–induced by malarial parasites. It also attributed promising antimalarial activity and afforded to improve the blood glucose and hemoglobin levels in treated mice. These research findings suggested the suitability of ellagic acid as a useful bioflavonoid for further study for the management of severe malaria pathogenesis.

## Introduction

According to the WHO, the incidence and death rates due to malaria have dropped down worldwide over the past 16 years; in 2018, there were 228 million new cases and 405,000 deaths from malaria. Still malaria is an important public health problem in many developing countries of the tropical and subtropical regions of the world, with high mortality in children and pregnant women (World Health Organization, 2019). Malaria is caused by *Plasmodium* parasites which are transmitted to humans through the bite of female mosquitoes of *Anopheles* species (Snow et al., 2005). Vaccination is the most effective method of preventing infectious diseases; however, producing an effective vaccine remains a challenge in malarial infection (Stanisic and Good, 2015). Current findings established that free radicals and its connection with oxidative stress in severe malaria can be responsible for several additional complications (Pabon et al., 2003; Becker et al., 2004). Clinical indications in severe malaria were found to be linked with the blood stage of infection with an excessive release of inflammatory cytokines (TNF-α, IL-6, and INF-γ) which contribute to further severity of infection such as organ damage and severe anemia (Saxena et al., 2016). Treatment of malaria has been complicated by the development of resistance to combination therapies based on artemisinin (Kumar et al., 2015).

Ellagic acid (EA) is a thermostable dilactone of hexahydroxydiphenic acid [2,3,7,8-tetrahydroxy-chromeno (5,4,3-cde)chromene-5,10-dione] with a molecular mass of 338.2 g/mol. EA is an important bioflavonoid present in several fruits, berries, and vegetables. It is also present as a primary constituent of various tannin-bearing antimalarial plants which are found in Africa (Vattem et al., 2005). EA has received considerable biomedical research attention due to their potent antioxidant activity and noticeable pharmacological effects on the prevention of various chronic pathological conditions linked with oxidative stress (Shakeri et al., 2018). EA possesses multiple health benefits including anti-inflammatory, antiviral, anticancer, antibacterial, hepatoprotective, cardioprotective, neuroprotective, gastroprotective, and antihyperlipidemic effects (Evtyugin et al., 2020). Considering the pharmacological importance of ellagic acid, several research groups are working on nanotechnology-related experimental approaches based on innovative oral drug carriers to improve its bioavailability (Ceci et al., 2020). In an attempt to evaluate the novel pharmacological activity of ellagic acid, we have explored the beneficial effect of EA on oxidative stress and inflammation-linked severe malaria pathogenesis in mice. The research findings of this study suggested the suitability of ellagic acid as a useful bioflavonoid for further study for the management of severe malaria pathogenesis.

## Materials and Methods

### Chemicals

**FIGURE 1:**
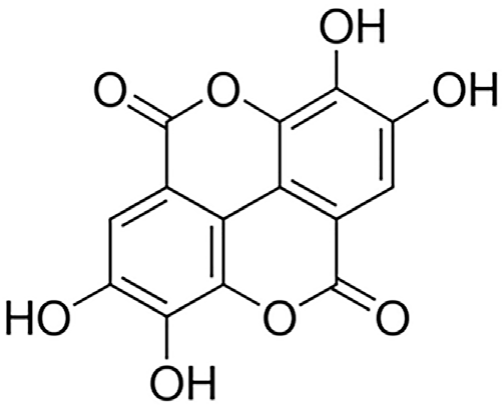
Chemical structure of ellagic acid.

Ellagic acid (Figure 1), chloroquine diphosphate, sodium chloride (NaCl), potassium dihydrogen phosphate (KH_2_PO_4_), LPS (*Escherichia coli* 055:B5), disodium hydrogen phosphate (Na_2_HPO_4_), DMEM, FBS, streptomycin, penicillin, potassium chloride, 2′,7′-dichlorofluorescein diacetate (DCFH-DA), trichloroacetic acid, thiobarbituric acid, pyrogallol, TMB, Evans blue, and dimethylsulfoxide (DMSO) were procured from Sigma-Aldrich, United States. Mouse- and rat-specific ELISA kits were purchased from BD Biosciences.

### *In Vivo* Study

#### Experimental Animals and Ethical Approval

Swiss albino (male, 18–22 g) mice were obtained from the institutional animal facility and acclimatized for 7 days under standard environmental conditions (12:12 dark-to-light cycle, 23 ± 2°C). After the termination of experiments, the experimental mice were euthanized by cervical dislocation after anesthetizing the animals using ketamine (80 mg/kg) and xylazine (10 mg/kg) as per the approved protocol (CIMAP/IAEC/2016-19/09). The vital organs of the experimental mice were immediately isolated for quantification of inflammation and oxidative stress markers. Animal experiments were performed as per the approved protocol by the Institutional Animal Ethics Committee (IAEC).

### Infection and Drug Treatment

Malaria was induced by giving intraperitoneal injection of *Plasmodium berghei* K-173–infected RBC’s into the experimental mice. The antimalarial activity was assessed using the method described by Knight and Peters (Kalia et al., 2015). One hour after infection, the experimental mice were orally treated with EA (5, 10, and 20 mg/kg) for 4 days consecutively. Carboxymethylcellulose (CMC) was used as a vehicle. Thin smears were prepared from blood of the infected mice and stained with Giemsa stain every alternate day from 4th day up to 28th day to calculate parasitemia by microscopic examination. Parasitemia was counted based on parasitized RBCs counted per 100 normal RBCs. Suppression of parasitemia after treatment was calculated by using the formula [(A-B)/A]100, where A denotes the mean percent parasitemia of the vehicle-treated mice and B denotes the mean percent parasitemia of the treated group. Survival of the infected experimental mice was observed up to the 28th day to calculate the percent survival and mean survival time.

### Quantification of Blood Glucose and Hemoglobin

On the peak day of parasitemia, blood glucose and hemoglobin were quantified to evaluate the possible effect of EA in malarial infection. Hemoglobin was quantified using Drabkin’s cyanmethemoglobin procedure and blood glucose using a glucometer.

### Quantification of Inflammatory Mediators

Blood was collected from each mouse for serum, and whole brain was isolated on the peak day of infection. Serum and brain homogenate were used for the quantification of pro-inflammatory cytokine production by using ELISA reagents.

### Quantification of Oxidative Stress Markers

To determine the effect of EA on organ damage caused by oxidative stress due to malaria infection in mice, a separate set of experiment was performed. The vital organs were harvested from the infected mice for oxidative stress marker quantification. In total, 10% (w/v) tissue homogenates were prepared and centrifuged at 5000 g for 10 min, and the collected supernatant was stored at -80°C immediately. The activity of superoxide dismutase was evaluated for its ability to inhibit pyrogallol autoxidation (Del Maestro and McDonald, 1985). The production of reactive oxygen species (ROS) was quantified using DCF-DA, a non-fluorescent cell-permeating compound, as described previously (Driver et al., 2000). Myeloperoxidase activity was quantified in tissue homogenates using TMB (3,3′5,5′ tetramethylbenzidine), as previously described (Suzuki et al., 1983). Lipid peroxidation was quantified by measuring the content of thiobarbituric acid reactive substances (TBARS) spectrophotometrically (Draper and Hadley, 1990).

#### Evans Blue Extravasation Assay

For Evans blue assay, 0.2 ml of 2% Evans blue solution in PBS was injected intraperitoneally into the infected mice to assess the blood–brain barrier permeability on the peak day of parasitemia. After 2 h, brain tissues were removed for dye extraction in 100% formamide. After 48 h, absorbance was measured at 620 nm and presented as micrograms of Evans blue stain per gram of brain tissue (Baptista et al., 2010).

### *In Vitro* Study

#### Quantification of ROS Generation and Pro-Inflammatory Mediators in C6 Glial Cells

C6 glial cells, procured from the National Centre for Cell Science (NCCS), Pune, India, were cultured using Ham’s F-10 media containing 10% FBS and placed in an incubator (5% CO2, 37°C). The cytotoxicity effect of EA was assessed using MTT assay on C6 cells, as described previously (Sharma et al., 2012). Intracellular ROS generation induced by H_2_O_2_ was calculated using DCFH-DA. The fluorescence intensity was measured with a spectrofluorometer (FLUO Star Omega, BMG Labtech), with an excitation wavelength of 485 nm and an emission wavelength of 520 nm. The intracellular reactive oxygen species production was also measured by flow cytometry (Vinoth et al., 2015). LPS-induced production of pro-inflammatory cytokines was quantified in the cell culture supernatant using rat-specific EIA reagents (Gupta et al., 2018).

### Statistical Analysis

Experimental data were shown as means ± SEM. The statistical significance of vehicle vs treatment groups was calculated using ANOVA, followed by Tukey’s multiple comparison tests. *p* < 0.05 was considered significant.

## Results

### Effect of EA on Parasitemia and Mortality in Malaria Pathogenesis

**FIGURE 2:**
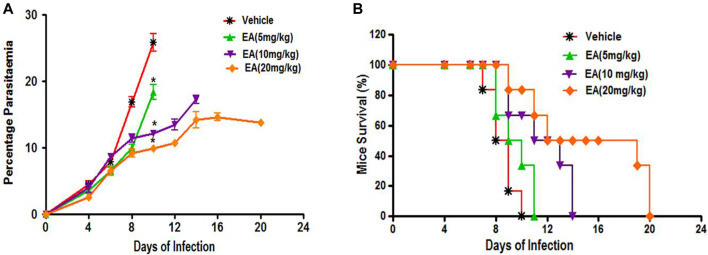
Effect of *EA* on antimalarial activity in P. berghei included malaria in mice **(A)** Percent Parasiteman **(B)** Percent Survival. Data are expressed as Mean ± SEM. *P < 0.05; *Vehicle vs Treatment; n = 6.

Oral treatment with EA at a dose of 5, 10, and 20 mg/kg showed a significant inhibition of parasitemia (*p* < 0.05) in comparison to the vehicle-treated infected mice in a dose-dependent manner (Figure 2A). Mean survival time was also significantly improved in the EA-treated group compared to the vehicle-treated infected group (Figure 2B).

### Effect of EA on Blood Glucose and Hemoglobin in Malaria Pathogenesis

**FIGURE 3:**
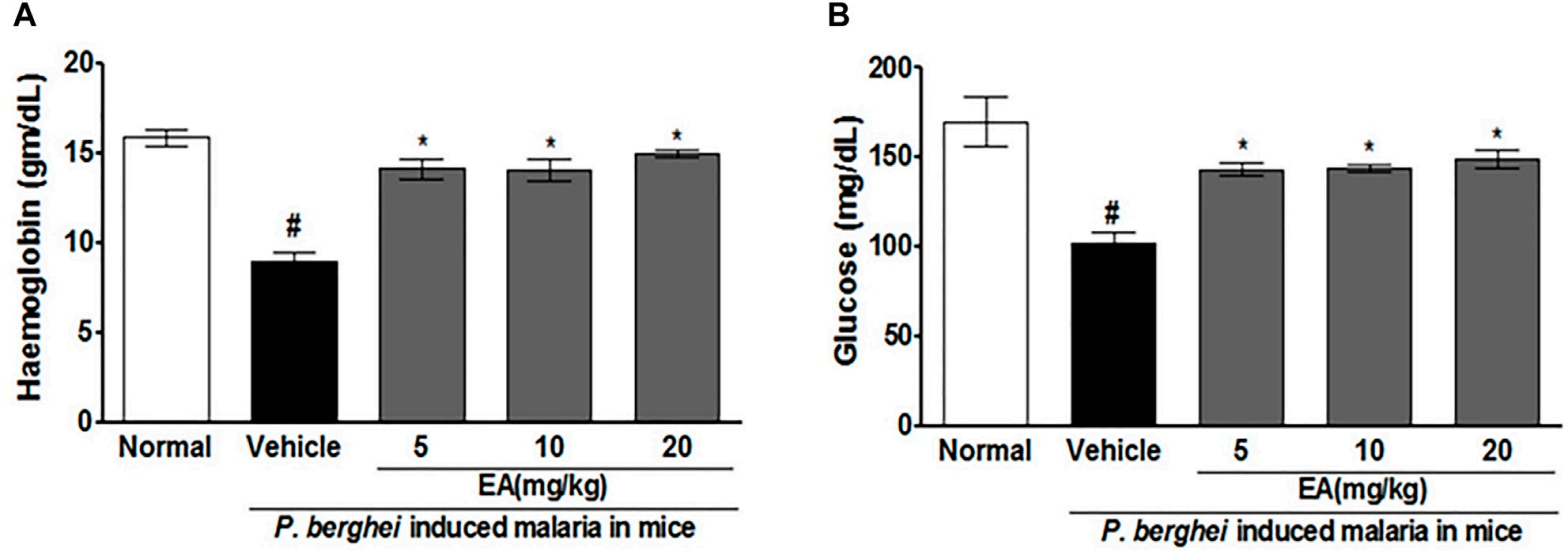
Effect of *EA* on haemoglobin and blood glucose level on peak day of parasitaemia in mice. **(A)** Haemoglobin **(B)** Blood glucose. Data are expressed as Mean ± SEM: *P < 0.05; #Normal vs vehicle treated; *Vehicle vs Treatment; n = 3.

Blood glucose and hemoglobin levels were found to be decreased significantly on the peak day of parasitemia in the vehicle-treated infected mice in compared to uninfected normal. Treatment with EA improved the blood glucose and hemoglobin in a dose-dependent manner (Figure 3).

Effect of EA on Oxidative and Antioxidative Markers in Malaria Pathogenesis

The MDA level, MPO activity, and ROS production were increased significantly (*p*˂0.05) in the brain, liver, and spleen of the vehicle-treated infected group in comparison to the uninfected normal group. EA treatment reduced the level of these oxidative stress markers compared with the vehicle-treated infected group. The SOD level was found to be significantly (*p*˂0.05) decreased in the vehicle-treated infected group compared to that in normal uninfected group, whereas EA-treated groups significantly increased the SOD level in comparison to the vehicle-treated infected group. The effect of EA treatment on the oxidative stress marker level in tissue homogenate is illustrated in Table 1.

**TABLE 1:** Effect of EA on oxidative and antioxidant stress marker levels in major organs affected by malaria infection. Data are expressed as mean±SEM; *P* < 0.05 considered statistically significant; *vehicle *vs* treatment group; # control *vs* vehicle group; *n* = 4.

	Groups	*P. berghei* infection	Treatment (mg/kg)	Pro-oxidative stress markers		Anti-oxidative stress marker	
				ROS (μmol DCF/ mg protein)	LPO (mM/mg protein)	MPO (U/mg protein)	SOD (U/mg protein)
Brain	Control	-		263.56 ± 12.58	0.100 ± 0.029	0.393 ± 0.008	1.709 ± 0.069
	Vehicle	✓	-	11166.61 ± 44.85^#^	3.104 ± 0.040^#^	1.714 ± 0.011^#^	0.267 ± 0.032^#^
	EA	✓	5	938.46 ± 14.45*	3.093 ± 0.029*	0.757 ± 0.001*	0.581 ± 0.042*
		✓	10	917.29 ± 16.59*	3.047 ± 0.035*	0.749 ± 0.006*	0.836 ± 0.024*
		✓	20	887.94 ± 34.68*	2.910 ± 0.052*	0.736 ± 0.016*	0.998 ± 0.016*
Liver	Control	-		280.47 ± 13.79	0.653 ± 0.03	0.322 ± 0.002	1.09 ± 0.03
	Vehicle	✓	-	1965.74 ± 18.91^#^	3.065 ± 0.09^#^	1.420 ± 0.019^#^	0.30 ± 0.02^#^
	EA	✓	5	1818.45 ± 8.50*	2.840 ± 0.05*	0.577 ± 0.004*	0.60 ± 0.04*
		✓	10	1704.07 ± 30.54*	2.444 ± 0.04*	0.550 ± 0.006*	0.65 ± 0.03*
		✓	20	11612.47 ± 13.80*	2.245 ± 0.08*	0.535 ± 0.044*	0.69 ± 0.09*
Spleen	Control	-		571.17 ± 38.63	0.533 ± 0.058	0.618 ± 0.027	1.206 ± 0.085
	Vehicle	✓	-	3926.17 ± 51.27^#^	3.463 ± 0.031^#^	1.625 ± 0.025^#^	0.437 ± 0.029^#^
	EA	✓	5	3878.22 ± 26.54*	3.433 ± 0.026*	0.762 ± 0.009*	0.640 ± 0.112*
		✓	10	3791.05 ± 80.28*	3.371 ± 0.109*	0.752 ± 0.002*	0.840 ± 0.081*
		✓	20	3496.46 ± 110.26*	3.209 ± 0.139*	0.734 ± 0.033*	1.156 ± 0.093*

### Effect of EA on Pro-Inflammatory Markers in Malaria Pathogenesis

**FIGURE 4:**
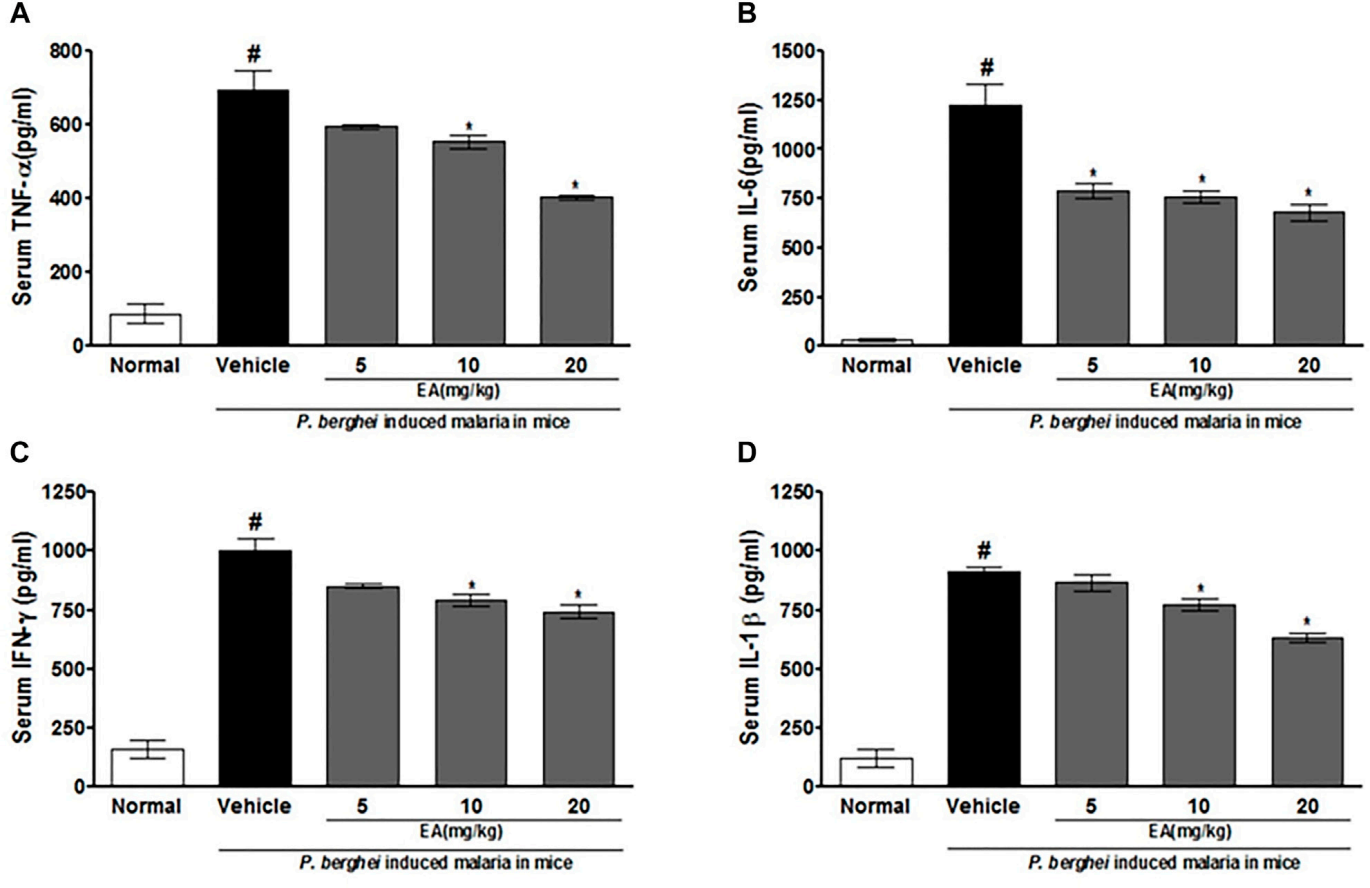
Effect of *EA* on production of pro-inflammatory cytokines TNF--α, IL-6, IFN-λ and IL-1β on peak day of parasitaemia in serum in *P. berghei* infected mice. Date are expressed as Mean ± SEM: *P* < 0.05; #Normal vs vehicle treated; *Vehicle vs treatment; *n* = 3.

**FIGURE 5:**
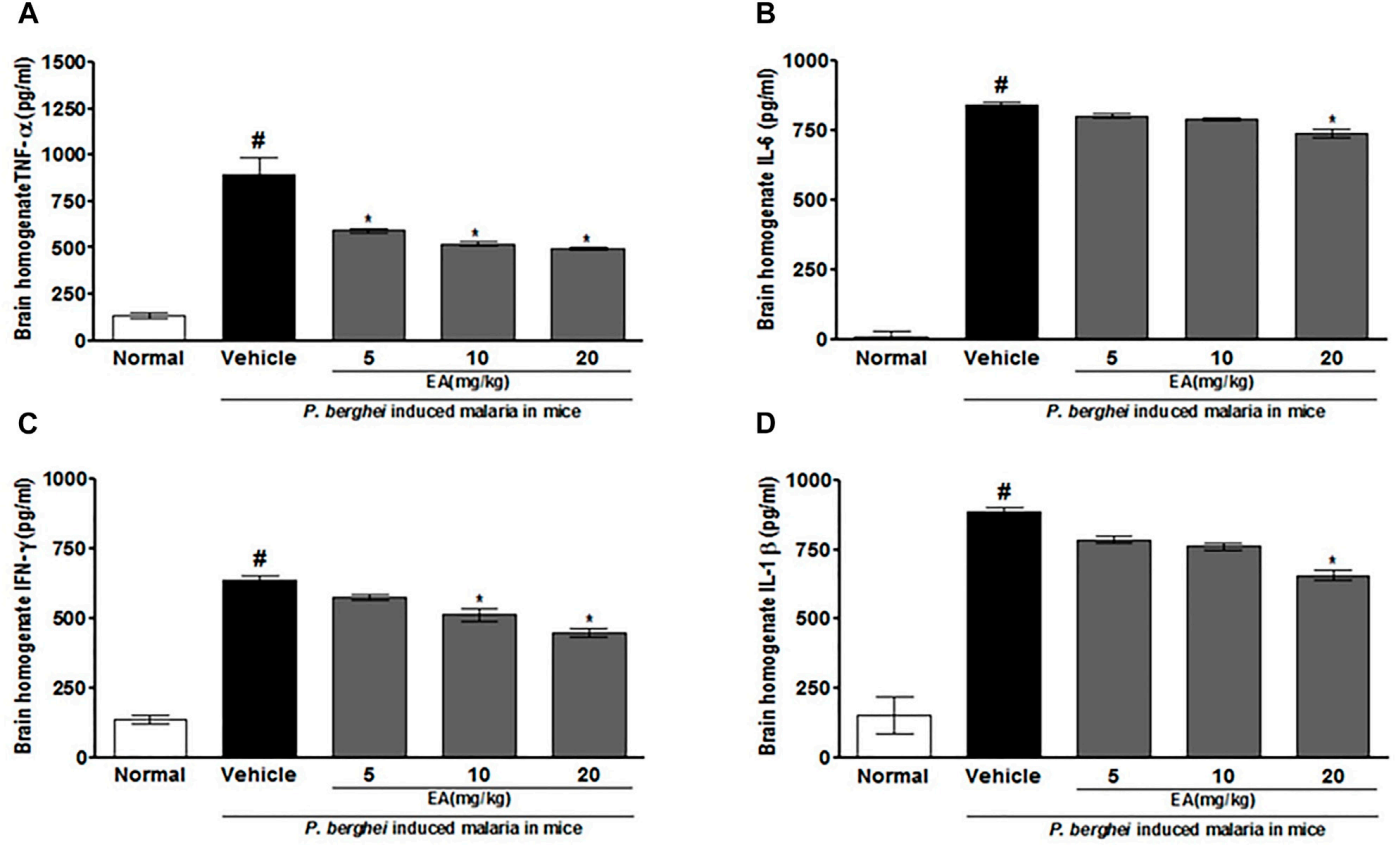
Effect of *EA* on production of pro-inflammatory cytokines TNF-α, IL-6, IFN-λ and IL-1β on peak day of parasitaemia in brain homogenate in P. berghei infected mice. Data are expressed as Mean ± SEM: *P* < 0.05; #Normal vs vehicle treated; *Vehicle vs treatment; n = 3. #Normal vs vehicle treated; *p < 0.05; *Vehicle vs treatment; *n* = 3.

The production of pro-inflammatory cytokines (TNF-α, IL-1β, IL-6, and IFN-γ) in serum and brain homogenate of the vehicle-treated infected mice was significantly increased compared to the normal group on the peak day of parasitemia. EA treatment showed a significant inhibition of pro-inflammatory cytokine production dose-dependently in malaria pathogenesis than in the vehicle-treated infected mice (Figures 4, 5).

Effect of EA on Blood–Brain Barrier Permeability in Malaria Pathogenesis

**FIGURE 6:**
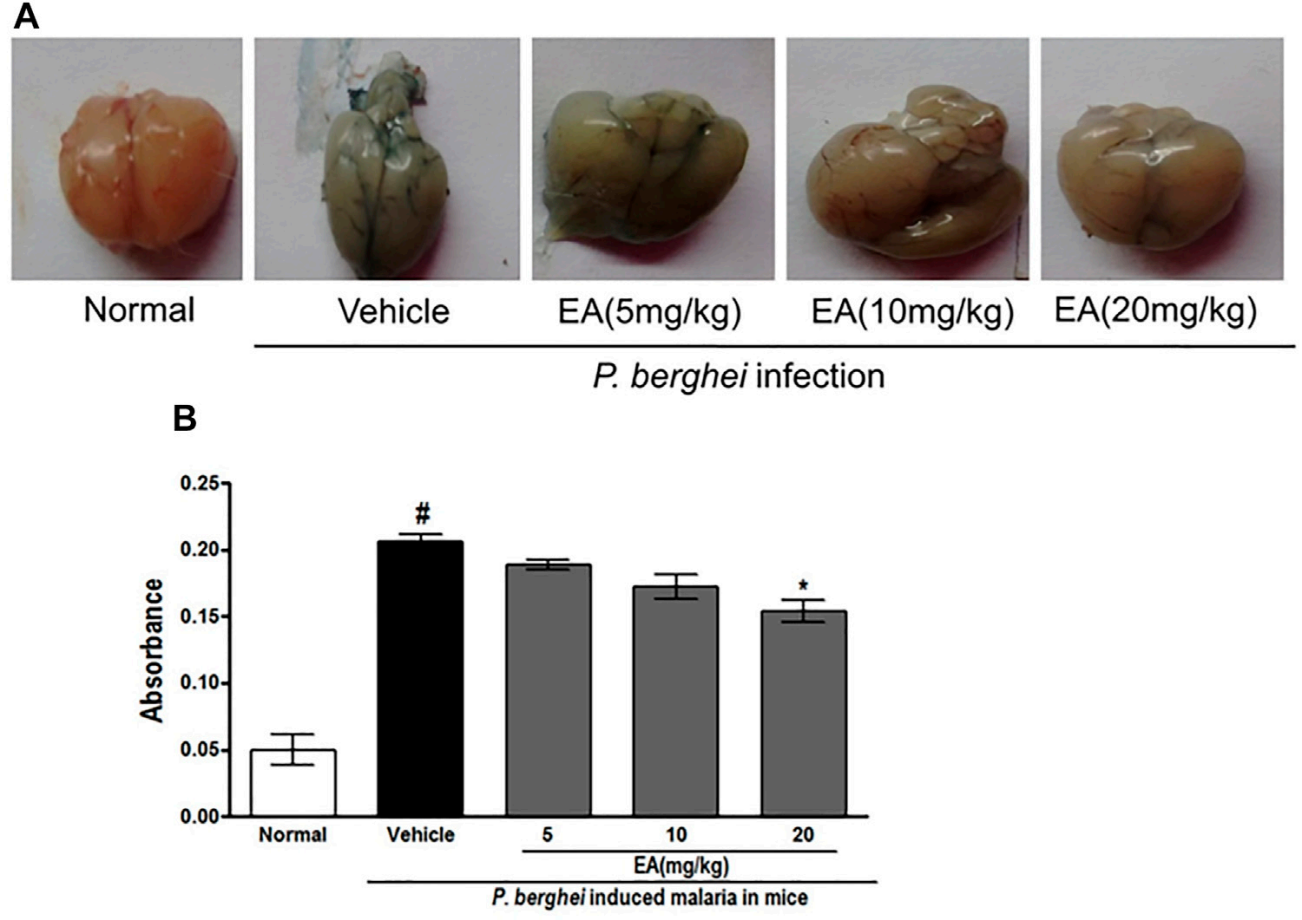
Vascular permeability of brains was assessed by Evans Blue injection on peak day of parasitaemia **(A)** Vascular leakage was assessed by the blue discoloration of the brain tissue of the various treated groups **(B)** Quantification of Evans Blue extravasation in formamide, absorbance was measured at 620 nm; *n* = 3. *P* < 0.05; #Normal vs vehicle treated; *Vehicle vs treatment.

An increase in the blood–brain barrier (BBB) permeability is a characteristic feature of severe malaria pathogenesis. Evans blue leakage assay showed that the normal mice have no disruption of BBB, whereas the brain isolated from the vehicle-treated infected mice has shown prominent dye accumulation, indicating the breakdown of the BBB during malaria pathogenesis. The brain isolated from the EA-treated mice has shown the significant (*p* < 0.05) lower staining of dye than that from the vehicle-treated infected mice (Figure 6).

### Cytotoxicity Profile of EA in Glial Cells

The effect of EA on cytotoxicity in C6 glial cells was studied using MTT assay. After EA treatment at 10, 30, and 100 μg/ml, percent live cell population was not significantly (*p* < 0.05) changed compared with normal untreated cells.

### Effect of EA on ROS Generation and Neuroinflammation in C6 Glial Cells

**FIGURE 7:**
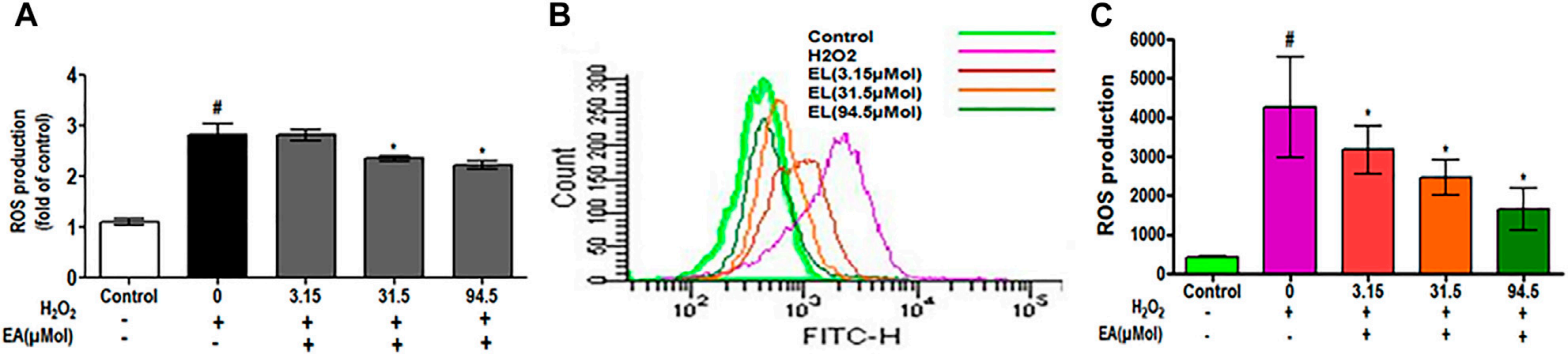
Effect of *EA* on H2O2-simulated ROS production using **(A)** spectrofluorometer and **(B, C)** flowcytometric analysis in C6 microglial cells. Data are expressed as Mean ± SEM; *p < 0.05 *treatment vs H_2_O_2_ treated cells. #*p* < 0.05 #control vs H_2_O_2_ treated cells; *n* = 3.

**FIGURE 8:**
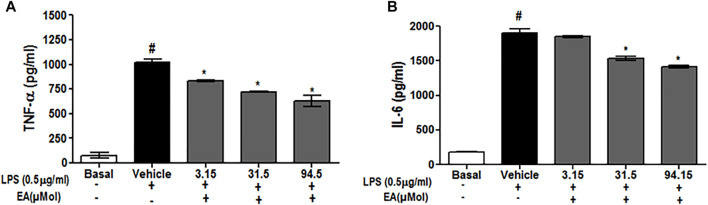
Effect of EA on production of pro-inflammatory cytokine **(A)** TNF-α and **(B)** IL-6 in C6 microglial cells. Data are expressed as Mean ± SEM; *P* < 0.05; #Normal vs Vehicle treated; *Vehicle vs treatment; n = 3.

To investigate the effect of EA on ROS generation, the spectrofluorometric and flow cytometric analyses were performed. The absorbance recorded spectrophotometrically showed that H_2_O_2_ alone significantly (*p* < 0.05) generated ROS in C6 glial cells after 1.5 h of incubation when compared with unstimulated cells. EA treatment (1, 10, 30 μg/ml) significantly attenuated H_2_O_2_-induced ROS generation in a dose-dependent manner when compared to H_2_O_2_-treated cells (Figure 7A). In agreement with the spectrophotometric observation, in the flow cytometric analysis, DCF-positive cells were found to decrease dose-dependently when treated with EA (1, 10, 30 μg/ml) (Figure 7B). Also, H_2_O_2_-induced cells exhibited higher DCF fluorescence intensities than the unstimulated cells. ROS generation was significantly (*p* < 0.05) decreased when the H_2_O_2_-induced cells were treated with EA (1, 10, 30 μg/ml) dose-dependently (Figure 7C). Experiments were performed on C6 glial cells stimulated with LPS to induce the production of pro-inflammatory cytokines. The production of pro-inflammatory cytokines (TNF-α and IL-6) in the cell culture supernatant was increased significantly in the LPS-stimulated cells compared with that in the normal cells. EA treatment (1, 10 and 30 μg/ml) exhibited a significant (*p* < 0.05) inhibition of pro-inflammatory cytokine production dose-dependently compared with the LPS-stimulated cells (Figure 8).

## Discussion

Malaria is a major public health problem in endemic countries with greater mortality in pregnant women and children, and severe malaria pathogenesis involves the series of host immune responses which eventually gets amplified, leading to severe organ injury and dysfunction (World Health Organization, 2019). Several research studies reported that malaria is a highly inflammatory condition characterized by acute periods of fever, headache, and nausea caused by *Plasmodium* infection of host red blood cells (Hunt, and Grau 2003; Togbe et al., 2007). Malaria-induced inflammatory cytokine storm circulating in the bloodstream of patients (Clark et al., 2008) is a critical pathological process which leads to severities like cerebral malaria, hypoglycemia, hyper-lactatemia, and acidosis (Schofield and Grau 2005) in malarial pathogenesis. Results of this study demonstrated that EA treatment is able to reduce parasitemia and increase the mean survival time in a dose-dependent manner in experimental malaria induced by *P. berghei*–infected RBCs in mice. Several findings suggest that plant-derived molecules exhibit antimalarial activity (Mohanty et al., 2013; Saxena et al., 2018; Gupta et al., 2018). Flavonoids are the natural molecules abundantly present in fruits and vegetables, showing beneficial effects against malarial pathogenesis (Puttappa et al., 2017; Amiri et al., 2018). Anemia and hypoglycemia are associated with severe malaria pathogenesis, which leads to mortality, especially in children and pregnant women (Boeuf et al., 2012). Oral treatment of EA is also able to restore hemoglobin and blood glucose in experimental malaria toward normal. Earlier published reports demonstrated that plant-derived natural molecules are able to restore hemoglobin and glucose levels in experimentally infected animals (Mohanty et al., 2015; Saxena et al., 2016). Oxidative stress due to malaria parasites if not checked by the host antioxidant mechanism can lead to oxidative damage in host tissues, which contributes to severe malarial pathogenesis (Becker et al., 2004). This study also revealed that EA treatment is able to protect from oxidative stress in vital organs during malarial pathogenesis. These findings are in agreement with the findings of recent reports that ellagic acid alleviates clozapine-induced oxidative stress and mitochondrial dysfunction in cardiomyocytes (Ahangari et al., 2020) and that plant-derived molecules are able to reduce the level of oxidative stress and improve the level of antioxidant enzymes in *P. berghei*–infected mice (Singh et al., 2017; Gupta et al., 2018). Results of this study reported a very high level of pro-inflammatory cytokine production (cytokine storms) in serum and the brain homogenate of *P. berghei* vehicle-treated infected mice on the peak day of parasitemia. EA treatment significantly inhibited the production of pro-inflammatory cytokines in serum and brain homogenates of malaria-infected mice in a dose-dependent manner when compared to the vehicle-treated infected mice. Cerebral malaria is the most severe form of infection with *Plasmodium falciparum* characterized by a highly inflammatory response (IFN-γ, IL-1β, TNF-α, iNOS, and IL-6), which contributes to severity of the disease (Angulo and Fresno, 2002). There are several reports concluding that standard antimalarial drugs (chloroquine and artemisinin) and other plant-derived molecules exert antimalarial activity by reducing parasitemia and also modulating the pro-inflammatory cytokines (Mohanty et al., 2015; Gupta et al., 2018). Severe acidosis, anemia, hypoxia, and renal and hepatic insufficiencies are associated with BBB dysfunction in malarial pathogenesis (Clark et al., 2004). In this study, the BBB integrity was restored by EA-treated mice as evident by reduced Evans blue extravasation. To further substantiate results related to the beneficial effect of EA treatment in malarial pathogenesis by promoting antioxidant and anti-inflammatory effects, we performed the additional experiments using C6 glial cells. EA significantly reduced the H_2_O_2_-induced ROS generation in C6 cells and LPS-induced inflammatory mediators (TNF-α and IL-6) in a dose-dependent manner without any cytotoxic effect. This finding also supports the previous finding which indicated that the neuroprotective role of ellagic acid is due to its antioxidant and anti-inflammatory effects on brain tissue (Wang et al., 2020).

## Conclusion

Taken together, the results of this study indicate that ellagic acid is able to alleviate severe malaria pathogenesis by reducing cytokine storms and oxidative stress induced by malaria parasites. It also attributed promising antimalarial activity and afforded to improve the blood glucose and hemoglobin levels in treated mice. These research findings suggested the suitability of ellagic acid as a useful bioflavonoid for further study for the management of severe malaria pathogenesis.

## Data Availability

The original contributions presented in the study are included in the article/Supplementary Material, and further inquiries can be directed to the corresponding author.

## Abbreviations

BSA, bovine serum albumin; CMC, carboxymethylcellulose; DMEM, Dulbecco’s modified Eagle medium; DMSO, dimethylsulfoxide; DCFH-DA, 2′,7′-dichlorofluorescein diacetate; DCF, dichlorofluorescein; EA, ellagic acid; ELISA, enzyme-linked immunosorbent assay; FBS, fetal bovine serum; H_2_O_2_, hydrogen peroxide; LPS, lipopolysaccharide; MTT, (3-(4,5-dimethylthiazol-2-yl)-2,5-diphenyltetrazolium); MPO, myeloperoxidase; MDA, malondialdehyde; PBS, phosphate-buffered saline; ROS, reactive oxygen species; SOD, superoxide dismutase; TBARS, thiobarbituric acid reactive substances; TBA, thiobarbituric acid; TMB, tetramethylbenzidine.
