# Supplementary figures and images for: Ameliorative Effects of Dietary Ellagic Acid Against Severe Malaria Pathogenesis by Reducing Cytokine Storms and Oxidative Stress
Source: Front Pharmacol. 2021 Dec 9;12:777400. doi: 10.3389/fphar.2021.777400 (PMC8717919; doi:10.3389/fphar.2021.777400)

## Slide 1
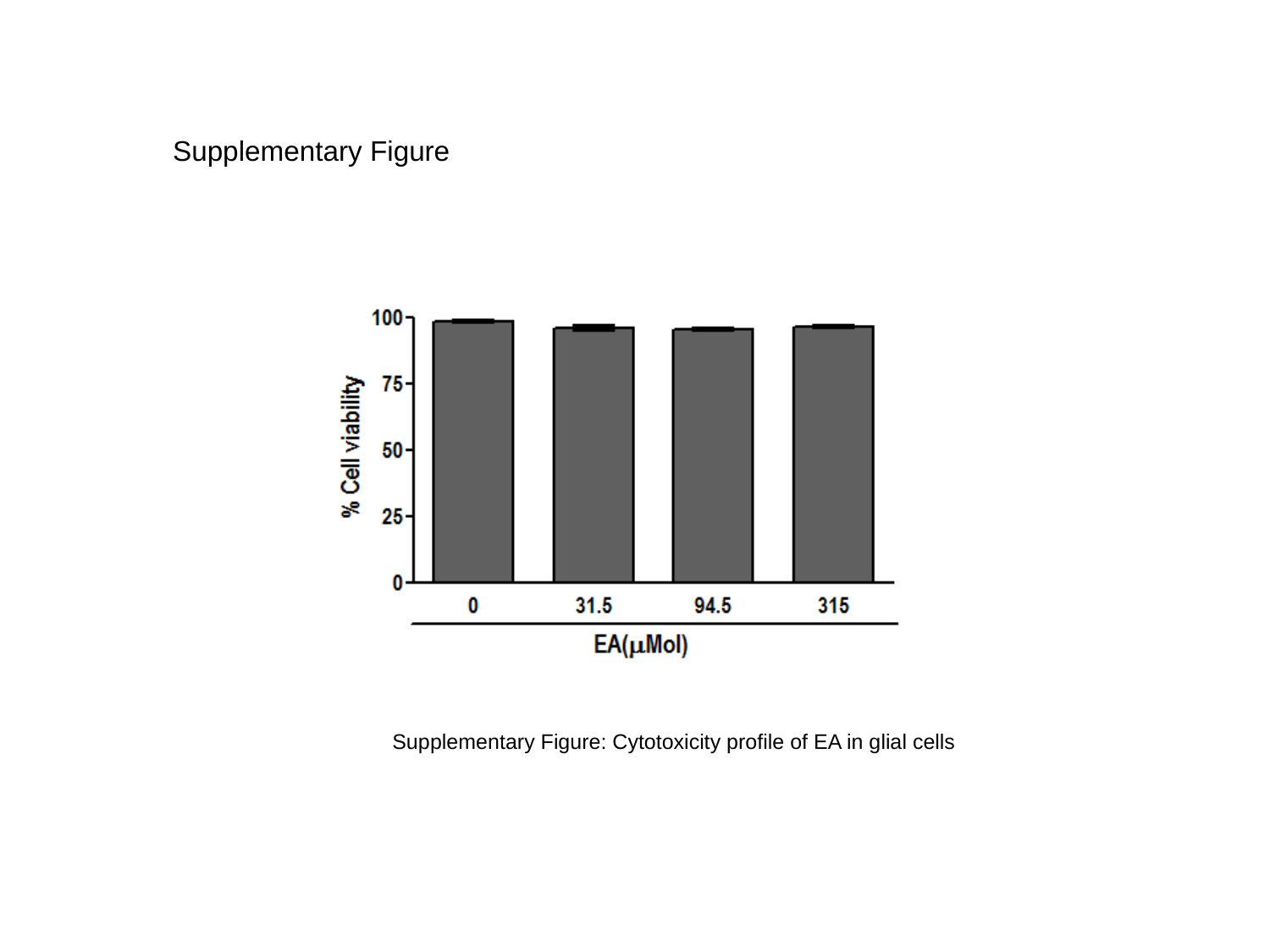

Supplementary Figure
Supplementary Figure: Cytotoxicity profile of EA in glial cells

Supplement: Supplementary file 1 [file Presentation2.PPT]

## Slide 1
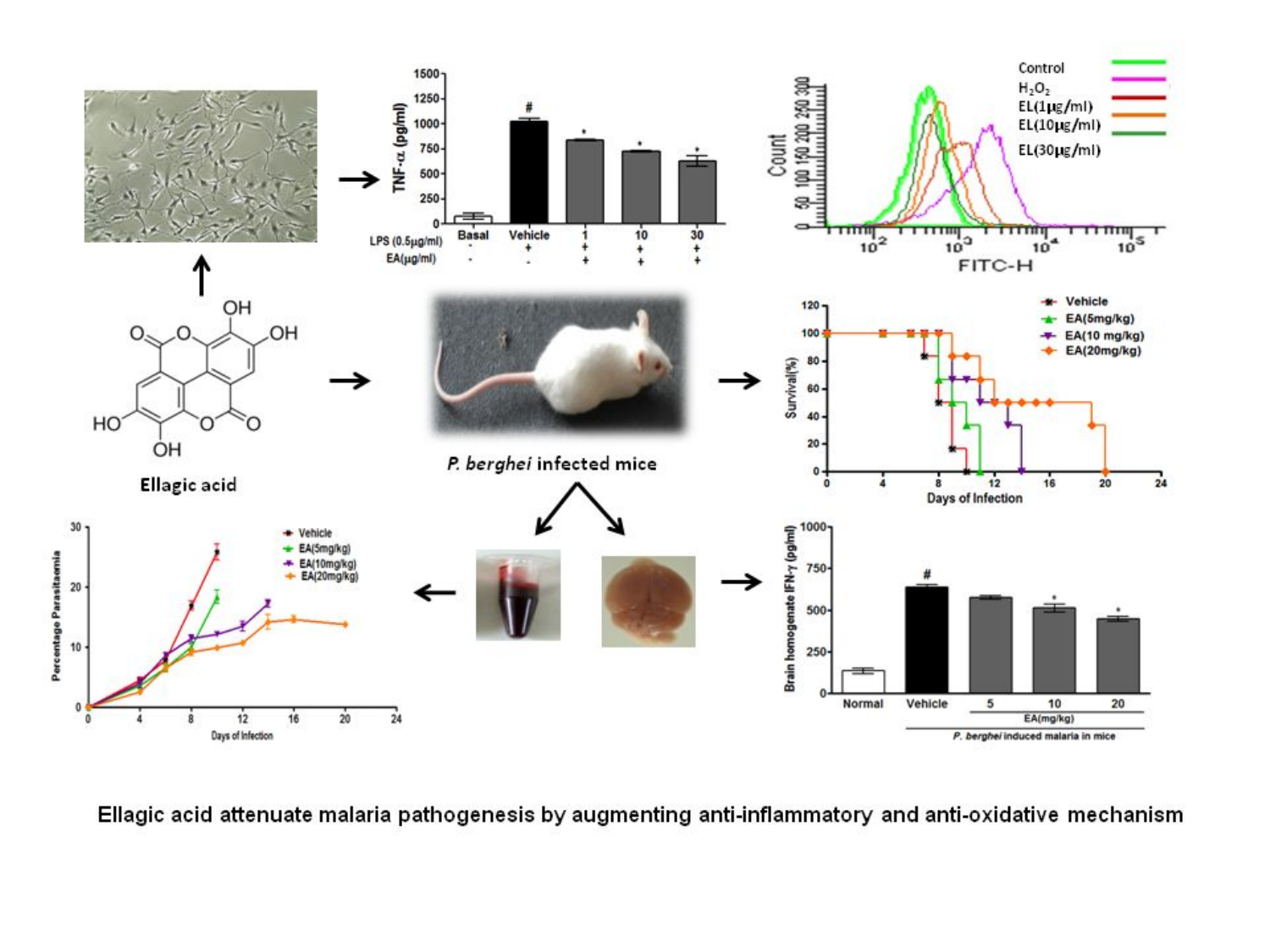

Supplement: Supplementary file 2 [file Presentation1.PPT]
